# Abrupt emissions reductions during COVID-19 contributed to record summer rainfall in China

**DOI:** 10.1038/s41467-022-28537-9

**Published:** 2022-02-18

**Authors:** Yang Yang, Lili Ren, Mingxuan Wu, Hailong Wang, Fengfei Song, L. Ruby Leung, Xin Hao, Jiandong Li, Lei Chen, Huimin Li, Liangying Zeng, Yang Zhou, Pinya Wang, Hong Liao, Jing Wang, Zhen-Qiang Zhou

**Affiliations:** 1 Jiangsu Key Laboratory of Atmospheric Environment Monitoring and Pollution Control, Jiangsu Collaborative Innovation Center of Atmospheric Environment and Equipment Technology, School of Environmental Science and Engineering, Nanjing University of Information Science and Technology, Nanjing, Jiangsu China; 2 Atmospheric Sciences and Global Change Division, Pacific Northwest National Laboratory, Richland, WA USA; 3 Frontier Science Centre for Deep Ocean Multispheres and Earth System and Physical Oceanography Laboratory, Ocean University of China, Qingdao, China; 4 Qingdao National Laboratory for Marine Science and Technology (QNLM), Qingdao, China; 5 Collaborative Innovation Center on Forecast and Evaluation of Meteorological Disasters/Key Laboratory of Meteorological Disaster, Ministry of Education, Nanjing University for Information Science and Technology, Nanjing, Jiangsu China; 6 Tianjin Key Laboratory for Oceanic Meteorology, Tianjin Institute of Meteorological Science, Tianjin, China; 7 Department of Atmospheric and Oceanic Sciences and Institute of Atmospheric Sciences, Fudan University, Shanghai, China

**Keywords:** Atmospheric chemistry, Atmospheric chemistry

## Abstract

Record rainfall and severe flooding struck eastern China in the summer of 2020. The extreme summer rainfall occurred during the COVID-19 pandemic, which started in China in early 2020 and spread rapidly across the globe. By disrupting human activities, substantial reductions in anthropogenic emissions of greenhouse gases and aerosols might have affected regional precipitation in many ways. Here, we investigate such connections and show that the abrupt emissions reductions during the pandemic strengthened the summer atmospheric convection over eastern China, resulting in a positive sea level pressure anomaly over northwestern Pacific Ocean. The latter enhanced moisture convergence to eastern China and further intensified rainfall in that region. Modeling experiments show that the reduction in aerosols had a stronger impact on precipitation than the decrease of greenhouse gases did. We conclude that through abrupt emissions reductions, the COVID-19 pandemic contributed importantly to the 2020 extreme summer rainfall in eastern China.

## Abstract (web summary)

In the summer of 2020, China experienced record rainfall and flooding. Here, the authors investigate the connection between the rainfall and the abrupt decrease in emissions due to Covid-19 and find that the emission decrease may have contributed to the rainfall.

## Introduction

In the early and middle summer of 2020, persistent extreme precipitation events occurred in eastern China. The accumulated rainfall has broken its 60-year record since 1961^1,2^. Eastern China, as one of the most urbanized and densely populated regions in the world, suffered severe damages and losses from this disaster. Extreme precipitation can induce severe stress on aquatic and terrestrial ecosystems, societal infrastructure, and socioeconomics^3,4^. From the perspective of climate variability, the strong Indian Ocean Dipole event in 2019^2^, the notably delayed withdrawal of the Meiyu-Baiu front caused by enhanced Arabian Sea warming^5^, and the sub-seasonal phase transition of the North Atlantic Oscillation^1^ were postulated as contributors of the unprecedented 2020 extreme summer rainfall in China. In addition to natural variability, human influence may also have a dramatic impact on extreme precipitations^6–8^.

The COVID-19 pandemic has caused massive disruptions of public life worldwide since February 2020^9,10^. To limit the spread of the disease, China adopted a series of restrictions, such as limiting nonessential activities and gatherings, temporarily shutting down industries and restricting transportations^11^. These dramatic restrictions are believed to have substantially reduced anthropogenic emissions of greenhouse gases (GHGs), aerosols and their precursor gases. Satellite sensors documented reductions in tropospheric column concentration of nitrogen dioxide and carbon dioxide emissions in China by 48% and 12%, respectively, after the outbreak of COVID‐19, compared with the pre-COVID period^12,13^.

Aerosols can affect clouds, precipitation, hydrological cycle and atmospheric circulation through microphysical as well as dynamical processes^14–18^. In the last four decades, summer precipitation over eastern-central China has decreased significantly, which has been reported to be closely related to the increase in aerosols over the region^19^. By cooling and stabilizing the lower atmosphere directly through scattering of solar radiation and indirectly by serving as cloud condensation nuclei, anthropogenic aerosols can reduce the occurrence of mesoscale convective systems by one-fifth to one-third, resulting in less precipitation in April over southern China^20^. Menon et al. argued that the increased summer flood in southern China and drought in northern China were mainly due to the absorbing black carbon aerosols, which heat the air, alter atmospheric stability, and disturb large-scale circulations and hydrologic cycle^21^.

Considering the important roles of aerosols in precipitation processes, this study investigates the impacts of COVID‐19 emissions reductions on regional extreme precipitation. We focus on the record summer precipitation in 2020 over eastern China where the most dramatic reductions of aerosols occurred. We find that the emissions reductions during COVID‐19 enhanced the summer atmospheric convection over eastern China. The resulting positive sea level pressure anomaly over the northwestern Pacific Ocean enhanced moisture convergence to eastern China and intensified the rainfall there. Our model results suggest that COVID-related reduction in aerosols could have contributed to about one-third of the observed 2020 extreme summer precipitation increase in eastern China.

## Results

### Unprecedented summer rainfall in eastern China

**Fig. 1:**
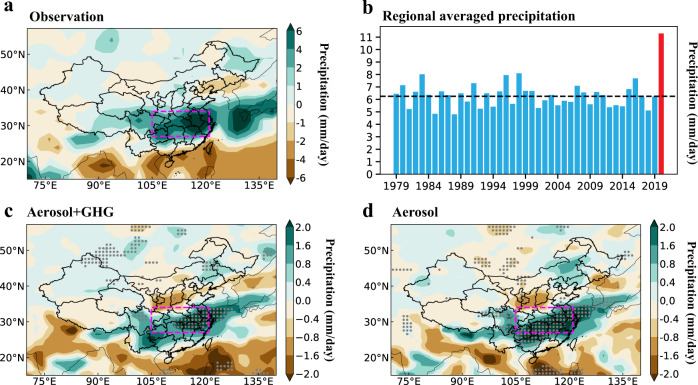
Extreme precipitation in China contributed by emissions reductions during COVID-19. **a** Spatial distribution of observed anomalies of June–July (JJ) mean precipitation rate (mm/day) over China in 2020 relative to the historical period of 1979–2019. **b** Time series of observed JJ mean precipitation rate (mm/day) in eastern China, marked by the red box (27–34°N, 105–121°E) in **a**. The black dashed line marks the JJ mean precipitation rate during 1979–2019, and the red bar is for 2020. The observations are obtained from the Global Precipitation Climatology Project (GPCP). Spatial distribution of changes in JJ precipitation rate (mm/day) for experiments **c** with COVID-19 forced reductions in aerosol emissions and greenhouse gases (GHGs) concentrations (referred to as Covid_All) and **d** with aerosol emission reductions alone (referred to as Covid_Aero), compared to the simulation experiment without emissions reductions (referred to as Baseline). The differences between Covid_All and Baseline are referred to “Aerosol+GHG” and the differences between Covid_Aerosol and Baseline are referred to “Aerosol”. The stippled areas in **c** and **d** indicate statistically significant differences at the 90% confidence level based on a two-tailed Student’s *t*-test.

China experienced a large increase in precipitation in June–July (JJ) of 2020, compared to the previous 41 years (1979–2019), with the largest increase of more than 6 mm day^−1^ over the Yangtze River Delta in eastern China (Fig. 1a). The precipitation in JJ averaged over the Yangtze River Delta in 2020 is 11.3 mm day^−1^, exceeding the 41-year average of 6.3 mm day^−1^ by 79% (Fig. 1b).

The abrupt emissions reductions due to the COVID-19 pandemic have been reported to exert a detectable impact on regional climate^22^. In this study, model sensitivity experiments performed using the Energy Exascale Earth System Model (E3SM) with COVID-19-induced reductions in aerosol emissions and GHGs concentrations (referred to as Covid_All) and with aerosol emission reductions alone (referred to as Covid_Aero) are compared to the control experiment without emission reductions (referred to as Baseline), each with 10 ensemble members, to explore the effects of abrupt emissions reductions on the 2020 extreme seasonal rainfall.

Including reductions in aerosol emissions and GHGs concentrations, the model remarkably reproduces the spatial pattern of observed precipitation anomalies over China. Statistically significant intensified precipitation in Covid_All, compared to Baseline, appears primarily over the Yangtze River Delta, where the JJ mean has an increase of 1.3 mm/day, which is about one-fourth (26%) of the observed increase (Fig. 1c). This result suggests that the abrupt emissions reductions contributed to the eastern China record summer precipitation in 2020. The simulated precipitation increase is mainly contributed by the decreases in aerosols and precursor emissions (Fig. 1d), which alone account for about one-third (32%) of the observed precipitation increase relative to the 1979–2019 summer mean over eastern China. Note that the precipitation responses to aerosol decreases vary quantitatively between different models shown below.

### Mechanisms of enhanced rainfall by emissions reductions

By disrupting human activities, the COVID-19 pandemic led to reduced aerosols and their precursor emissions^23^ (Supplementary Fig. 1), which caused a decrease in near-surface PM_2.5_ concentrations^24^. Compared to JJ in 2019, observations showed that PM_2.5_ concentrations in 2020 were largely reduced in the Yangtze River Delta and Pearl River Delta, with a maximum decrease of up to 35% (Supplementary Fig. 2). The model reasonably reproduces the overall spatial distribution of changes in near-surface PM_2.5_ concentrations due to COVID-19 related emissions reductions, with less discernable changes (even increases) in northern China and more substantial decreases in southern China. Increases in rainfall can also remove aerosols from the atmosphere in eastern China, intensifying the decreases in aerosol concentrations^25^. Satellite retrieved aerosol optical depth (AOD) also supports the modelled decrease in aerosols over eastern China during the COVID-19 pandemic (Supplementary Fig. 3).

**Fig. 2:**
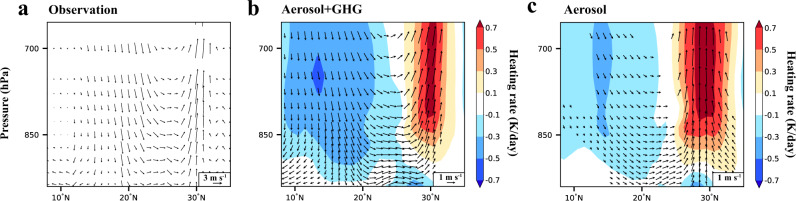
Changes in vertical profiles of heating rates and winds due to COVID-19 emissions reductions. **a** Changes in observed June–July mean meridional wind (m s^−1^, vectors) and pressure velocity (Pa s^−1^, vectors) multiplied by –100, averaged over 105–125°E in 2020, relative to the historical average over 1979–2019 from ERA5 reanalysis data. Panels **b** and **c** are the changes in June–July mean meridional wind (m s^−1^, vectors), pressure velocity (Pa s^−1^, vectors) multiplied by −100 and atmospheric heating rate (K day^−1^, shaded) from Covid_All and Covid_Aero, respectively, compared to Baseline. Only vertical circulation changes that are statistically significant at the 90% confidence level are shown in **b** and **c**.

The decreases in aerosols during the pandemic resulted in anomalous atmospheric heating over 25°−35°N in eastern China and downwind areas (Fig. 2). The simulated heating rates are higher than 0.7 K day^−1^ in the emission reductions simulations relative to Baseline, which increased atmospheric instability and produced an anomalous ascending flow over eastern China. The enhanced convection over eastern China strengthened the dynamical condition for extreme precipitation. Aerosol reduction is identified as the main reason for the ascent since it appears in both emissions reductions simulations. Due to a compensating cooling effect associated with the decrease in GHGs concentrations in Covid_All, the anomalous heating and ascent therein (Fig. 2b) are not as strong as those in Covid_Aero (Fig. 2c), explaining the stronger impact of aerosol reductions alone on the 2020 extreme summer precipitation in eastern China than the combined effect of aerosols and GHGs (Fig. 1c, d). The aerosol reduction-induced instability and vertical circulation change in eastern China were also reported in previous studies^26,27^. The reduced aerosol loadings can significantly enhance the occurrence of local convective systems^14^, causing the release of additional latent heat at higher altitudes through intensified ascending motions^20^. Therefore, profound positive heating anomalies can be observed at higher altitudes above 850 hPa, rather than near the surface (Fig. 2b, c), while the weak local negative heating anomalies may be attributed to the cooling induced by increases in precipitation and/or the adiabatic expansion. The anomalous ascent over land also resembles the observed pattern but with a weaker strength (Fig. 2a), supporting the hypothesis that aerosol reductions during COVID-19 contributed to the favorable atmospheric condition conducive to extreme precipitation in eastern China.

The anomalous heating over land intensifies the summer land-sea air temperature difference and the circulation between eastern China and the South China Sea/Philippine Sea, as also presented in ERA5 reanalysis (Fig. 2a). The anomalous subsidence over the ocean induced by reductions in both aerosols and GHGs is more intense than that caused by the reduction in aerosols alone, due to the additional atmospheric cooling associated with GHGs reductions. The anomalous subsidence can lead to positive anomalies of sea level pressure (SLP) without inducing changes to SST^27^, which further affect atmospheric dynamical processes and precipitation^28–30^.

**Fig. 3:**
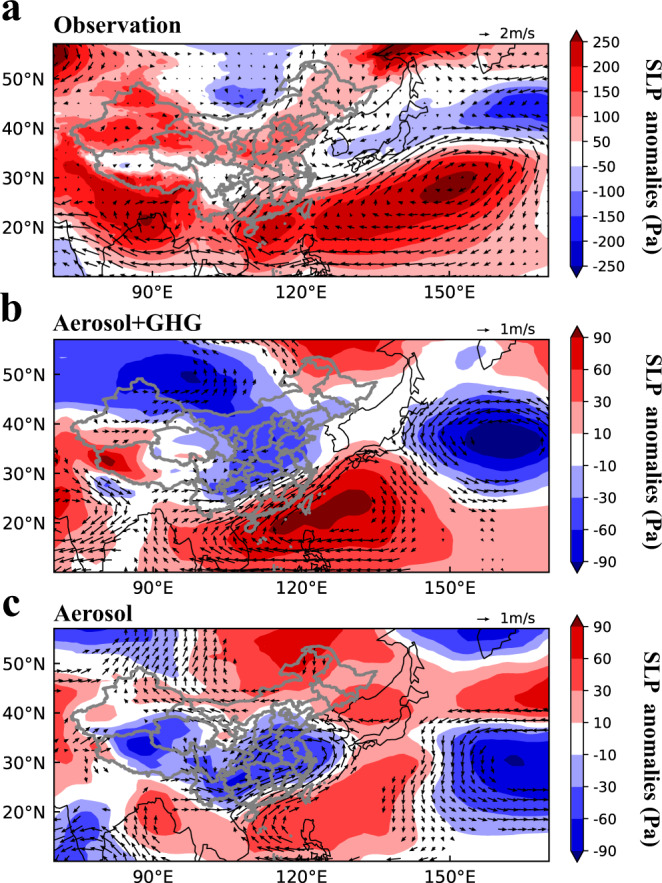
Atmospheric circulation and sea level pressure (SLP) anomalies. **a** The observed anomalies of June–July mean SLP (Pa, shaded) and 850 hPa winds (m s^−1^, vector) in 2020, relative to the historical average over 1979–2019. The anomalies of meteorological fields from 1979 to 2020 are obtained from ERA5 reanalysis data. Panles **b** and **c** are same as **a** but for the simulated SLP and circulation changes in Covid_All and Covid_Aero, respectively, compared to Baseline. Only circulation changes that are statistically significant at the 90% confidence level are shown in **b** and **c**.

In ERA5 reanalysis, positive SLP anomalies off the southeast coast of China covered a large fraction of the Northwest Pacific during the summer of 2020, compared to the climatological average of the previous four decades (Fig. 3a), intensifying the Western Pacific subtropical high (WPSH). The anomalous southwesterlies along the northwestern edge of the intensified WPSH brought moist air from the South China Sea to eastern China, leading to enhanced rainfall in 2020. With the abrupt reductions in aerosols and GHGs, Covid_All realistically reproduces the anomalous atmospheric circulation and SLP patterns, with magnitudes equivalent to about one-third to half of the observed values (Fig. 3b). The aerosol-induced atmospheric circulation and SLP changes (Covid_Aero) in Fig. 3c also resemble the observed spatial patterns and are consistent with the signature in Covid_All, although the SLP change is weaker than that in COVID_All, indicating that both aerosols and GHGs can explicate the observed anomalous positive SLP over the ocean.

**Fig. 4:**
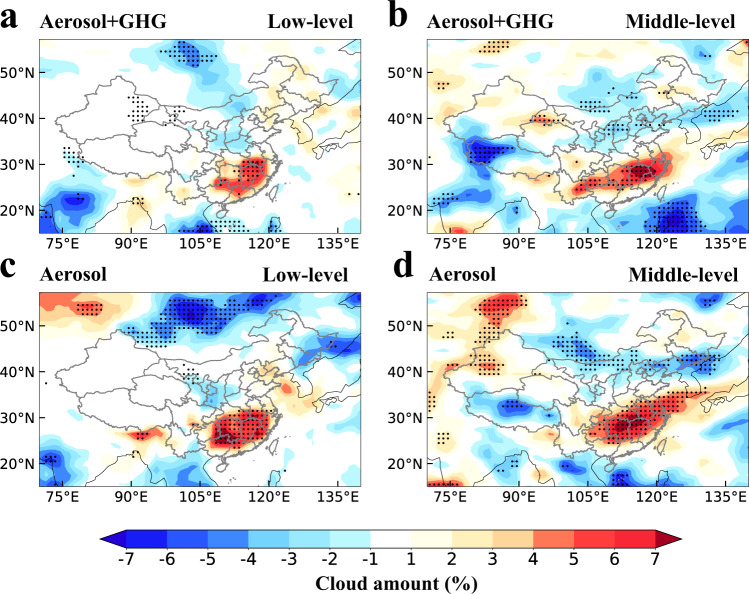
Changes in cloud amount due to COVID-19 emissions reductions. Spatial distribution of changes in June–July low-level (1000–700 hPa; **a**, **c**) and middle-level (700–400 hPa; **b**, **d**) cloud amount (%) for Covid_All (**a**, **b**) and Covid_Aero (**c**, **d**) compared to Baseline. The stippled areas indicate statistically significant differences at the 90% confidence level based on a two-tailed Student’s *t*-test.

The anomalous anticyclone of the intensified WPSH associated with the positive SLP anomaly results in an enhancement of moisture advection towards eastern China and the associated moisture convergence (Supplementary Fig. 4), and subsequently changes clouds and produces the favorable moist condition for extreme precipitation. Satellite observations show spatially extensive increases in the total cloud cover during the summer of 2020 compared to 2019, especially in eastern China, with a maximum increase of 12–16% (Supplementary Fig. 5a). The simulated responses in the total cloud cover are highly consistent with the observations in both spatial distribution and magnitude (Supplementary Fig. 5b, c). A robust and statistically significant increase in the low-level cloud cover by 1–8% is found in both the Covid_All and Covid_Aero experiments compared to Baseline over eastern China (Fig. 4a, c). Similar changes are shown in the middle-level cloud cover in the 2020 summer (Fig. 4b, d).

The increased amount of thick clouds during the COVID-19 pandemic along with the anomalous ascending flow and moisture convergence led to an increase in both convective and large-scale precipitation (Supplementary Fig. 6) that explains one-third of the 2020 summer rainfall increase in eastern China. This finding is supported by Ding et al.^31^. who reported the role of moisture convergence and ascent in the 2020 extreme precipitation, although they attributed the circulation anomalies to a significant quasi-biweekly oscillation of East Asian monsoon caused by natural variability. Although both aerosols and GHGs reductions increase SLP over the ocean and provide favorable moist conditions, aerosol reductions dominate the ascent over land and exert the primary influence on the enhanced extreme precipitation in the experiments (Fig. 1c, d).

Results from a single model may not be representative. In the Covid multi-Earth system model intercomparison project (CovidMIP^32^), almost all models (6 out of 7 available models including E3SM) reproduced the increased precipitation over the Yangtze River Delta in eastern China (Supplementary Fig. 7), although the location and magnitude of the maximum increase vary in eastern China. This indicates that the E3SM simulated enhanced summer precipitation over eastern China by the abrupt emissions reductions during COVID-19 (Supplementary Fig. 8) is less likely a model-dependent feature. Note that the only exception (CanESM5) among the models is also the outlier in simulating the global and Asian AOD changes in response to emissions reductions among the CovidMIP models^32^. These diverging results appear to be related to the unexpected changes in natural dust emissions due to the simulated changes in near-surface winds in CanESM5. The strong changes in dust loading can induce an additional impact on atmospheric heating and dynamical processes, which complicate the circulation changes associated with reductions in anthropogenic aerosols and GHGs. Notably, the emission reduction-induced instability and vertical circulation change in eastern China also exist in other CovidMIP models (Supplementary Fig. 9), supporting the proposed mechanism based on E3SM results. However, we also note that different models have different responses in magnitude related to different physical and chemical processes of aerosols and the complexity of aerosol effects on climate, causing uncertainties in the precipitation changes related to the emissions reductions.

## Discussions

Record summer precipitation over eastern China in 2020 caused severe flooding and significant losses. This study investigates the impact of COVID-19-induced abrupt emissions reductions on the unprecedented summer precipitation in China. We find that aerosol reductions are a crucial factor in enhancing the extreme summer precipitation during the COVID-19 pandemic, explaining about one-third of the observed extreme rainfall increase over eastern China during that summer, although the magnitudes of precipitation responses vary among models. This study underscores the atmospheric convection and large-scale circulation changes in response to the spatially expansive aerosol reductions that increased precipitation over a prolonged period and resulted in flooding in a large region of China. Contrasting this mechanism with the aerosol effect on individual storms^33^ is important for improving the prediction of extreme precipitation and flood risk management.

Due to the sudden emissions reductions, the GHGs and aerosol effects were mainly mediated through fast climate responses^34,35^, including the changes in atmospheric convection and large-scale circulation. Although human activities and emissions were largely resumed in summer 2020, the emissions reductions were profound during the early months of 2020 in China. The impact of emissions reductions in the early months could have ramifications persisting across seasonal timescale through oceanic feedbacks^36,37^, adding to the immediate atmospheric responses to summertime emissions reductions.

Anthropogenic aerosol emissions have been reducing in China in recent years due to emission control to improve air quality. Why was the precipitation response to the COVID-induced emissions reductions in eastern China during 2020 so different from the precipitation response to reduced emissions in previous years? Notably, emissions were reduced dramatically in early 2020 when the COVID-19 pandemic emerged suddenly, causing an immediate and abrupt change in various components of the climate system. Such sudden change of the climate system could be very different from changes in response to continuous but gradual policy-driven emissions reductions. Furthermore, unlike GHGs, aerosol changes and associated effects are strongly nonlinear and non-uniform, so even small additional aerosol reductions during the pandemic could potentially lead to a more dramatic change in the Earth system^38^.

Some uncertainties in this study should be noted. Because the simulated aerosol concentrations in China are underestimated in the model in part due to the lack of treatment of nitrate and ammonium aerosols, strong wet scavenging, and less transformation from gas to particles^39^, the impact of aerosol emissions reductions on extreme precipitation could have been underestimated. In addition to the aerosol impacts on meteorological fields, studies also found that unfavorable atmospheric conditions could intensify aerosol pollution during the COVID-19 pandemic over the North China Plain^40,41^, which possibly explains the observed PM_2.5_ increases in northern China (Supplementary Fig. 2).

Both scattering and absorbing aerosols can influence precipitation through microphysical and dynamical processes. By reducing absorbing black carbon (BC) aerosol emissions alone in a sensitivity experiment using the Community Earth System Model version 1 (CESM1^42^), with scattering aerosols and GHGs unchanged, the precipitation enhancement over eastern China shown in Covid_All reverses to precipitation suppression (Supplementary Fig. 10), suggesting that the reductions in scattering aerosols, rather than absorbing aerosols, are likely the key factor contributing to the record rainfall.

Reductions in aerosol loading over eastern China can be due to either the reduced local emissions or less aerosol transport from South and Southeast Asia associated with emissions reductions in these regions. Using an explicit aerosol source tagging technique implemented in the Community Atmosphere Model (CAM5-EAST^43–46^), we found that emissions from South and Southeast Asia only account for 8.3% (±2.6%) of sulfate and 6.3% (±1.4%) of BC column burden in June–July over eastern China, while China domestic emissions are the dominant contributor to the sulfate (71.7% ± 7.9%) and BC (88.6% ± 2.3%) burden over 1980–2018. Therefore, the decreases in emissions from South and Southeast Asia are not likely the cause of the decrease in aerosols over eastern China and the enhanced extreme precipitation in summer 2020. However, the aerosol reductions in South Asia may perturb Asian rainfall by influencing the South Asian summer monsoon, which is worthy of further in-depth analysis and investigation.

In previous studies, Liu et al.^1^. postulated the sequential warm and cold Meiyu front regulated by the North Atlantic Oscillation as the cause of the record summer precipitation in 2020, while Zhou et al.^2^. indicated that the strong Indian Ocean Dipole event in 2019 was the main contributor. Although such internal variabilities likely played an important role in the extreme precipitation in 2020, the impact of anthropogenic factors should not be ignored. Neither of the aforementioned studies considered the effect of abrupt human influence with a footprint of substantial spatial scale and magnitude. Interactions between aerosols, convection, atmospheric circulation and clouds are important processes to consider in weather and climate predictions. The regional and temporary emissions reductions that have occurred during the pandemic may continue to occur in the future, though likely of smaller magnitude, as the society adapts and evolves. This may lend opportunities to further advance understanding of how weather and climate respond to short-term emission perturbations. This study shows that such response includes changes in extreme weather events with unanticipated consequences.

## Methods

### COVID-19 emissions reductions

Reduced human activities during the COVID‐19 lockdown and restrictions in 2020, especially less traffic on the road, led to fewer emissions nationwide in China. The estimated emissions reductions follow Forster et al.^23^. Overall, COVID‐19 resulted in reductions in anthropogenic emissions of sulfur dioxide (SO_2_), black carbon (BC), and organic carbon (OC) by 9–15%, 3–6%, and 0–3%, respectively, over eastern China in June–July 2020, compared to the SSP (Shared Socioeconomic Pathways) 2–4.5 baseline emission scenario. In the year 2020, the largest decreases took place in February, which was in line with the full lockdown in eastern China that started from January 23rd to 25th and remained until the end of February. In the meantime, due to the severity of the outbreak of COVID‐19, major manufacturing activities and pollutant emissions from various industrial sectors did not fully recover until the end of 2020. The global and annual GHGs concentrations of CO_2_ (carbon dioxide), CH_4_ (methane), and N_2_O (nitrous oxide) decreased from 412.46 ppm (parts per million), 1901.9 ppb (parts per billion) and 331.95 ppb to 412.06 ppm, 1900.2 ppb and 331.93 ppb, respectively, in 2020 due to the COVID-19 emissions reductions provided by the CovidMIP input data^32^.

### Model description and experimental setup

We conduct model simulations using the state-of-the-science Energy Exascale Earth System Model version 1.1 (E3SMv1.1^47,48^), which participated in the Covid multi-Earth system model intercomparison project (CovidMIP^32^). E3SMv1.1 is a fully coupled model with a dynamical ocean component. The default atmospheric model configuration includes 72 vertical layers at a spatial resolution of ~1°. The model simulates major anthropogenic aerosol species, including sulfate, black carbon (BC), primary organic matter (POM) and secondary organic aerosol (SOA) along with natural dust and sea salt aerosols using a four-mode modal aerosol module (MAM4). Details of aerosol‐related treatments are described and evaluated in Wang et al.^49^.

The aerosols and their precursor gas emissions and GHGs concentrations used in the baseline simulation are based on the CMIP6 (the Coupled Model Intercomparison Project Phase 6) SSP 2–4.5 emission scenario, referred to here as the Baseline experiment. To characterize the short-term influence of COVID‐19-induced aerosol changes, ensemble simulations (referred to as Covid_Aero), run in parallel with Baseline, start with the aerosol emission reduction mentioned above from 1 January 2020. In order to fully reflect the COVID‐19 influences, another set of simulations (referred to here as experiment Covid_All), is conducted with a similar configuration to the experiment Covid_Aero but with the GHGs concentrations reductions included as well. The E3SMv1.1 does not simulate carbon cycle and CO_2_ online, but rather takes GHGs concentrations provided by the CovidMIP input data. Because the global mean and regional climate signals caused by the COVID-19 reductions of emissions is weak relative to the climate system internal variability, ten ensemble members branched from 1 January 2020, initiated with a tiny perturbation in atmospheric temperature, are launched for each experiment to detect the signal of climate change from the random noise of internal variability following CovidMIP protocol. The effects of abrupt emissions reductions on the 2020 seasonal extreme rainfall are analyzed by comparing Covid_All/Covid_Aero and Baseline ensemble simulations, of which the members are initiated with identical ocean states. By differing the two simulations, the impacts from initial oceanic states are minimized.

## Supplementary information

Supplementary Information

Peer Review File

## Data Availability

The daily precipitation data covering 1979–2020 are publicly available from the Global Precipitation Climatology Project (GPCP, https://www.ncei.noaa.gov/data/global-precipitation-climatology-project-gpcp-monthly/access/). The monthly aerosol optical depth (AOD) and total cloud fraction observations during 2019–2020 can be derived from the Moderate Resolution Imaging Spectroradiometer data (MODIS, https://ladsweb.modaps.eosdis.nasa.gov/search/). The surface PM_2.5_ (particulate matter less than 2.5 μm in diameter) observations over China in 2019 and 2020 can be obtained from the China National Environmental Monitoring Center (CNEMC, 10.5281/zenodo.5775330). The anomalies of meteorological fields from 1979 to 2020 can be obtained from ERA5 reanalysis data (https://www.ecmwf.int/en/forecasts/datasets/reanalysis-datasets/era5). The CovidMIP multi-model results can be found at https://esgf-node.llnl.gov/search/cmip6/. The processed modeling data are available at 10.5281/zenodo.5775314.

## Code availability

The source code of E3SM is available at https://github.com/E3SM-Project/E3SM. The codes that support the findings of this study are routinely and commonly used in the community, which are fully available from the first author (yang.yang@nuist.edu.cn) upon reasonable request.
