# Supplementary material for: Abrupt emissions reductions during COVID-19 contributed to record summer rainfall in China
Source: Nat Commun. 2022 Feb 18;13:959. doi: 10.1038/s41467-022-28537-9 (PMC8857220; doi:10.1038/s41467-022-28537-9)
Supplement: Supplementary file 1 — Supplementary Information [file 41467_2022_28537_MOESM1_ESM.pdf]

Supplementary material for "**Abrupt emissions reductions during COVID-19 contributed to record summer rainfall in China**"

Yang Yang[1*,#], Lili Ren[1,#], Mingxuan Wu[2], Hailong Wang[2*], Fengfei Song[2], L. Ruby Leung[2], Xin Hao[3], Jiandong Li[1], Lei Chen[1], Huimin Li[1], Liangying Zeng[1], Yang Zhou[1], Pinya Wang[1], Hong Liao[1], Jing Wang[4], Zhen-Qiang Zhou[5]

[1]Jiangsu Key Laboratory of Atmospheric Environment Monitoring and Pollution Control, Jiangsu Collaborative Innovation Center of Atmospheric Environment and Equipment Technology, School of Environmental Science and Engineering, Nanjing University of Information Science and Technology, Nanjing, Jiangsu, China

[2]Atmospheric Sciences and Global Change Division, Pacific Northwest National Laboratory, Richland, Washington, USA

[3]Collaborative Innovation Center on Forecast and Evaluation of Meteorological Disasters/Key Laboratory of Meteorological Disaster, Ministry of Education, Nanjing University for Information Science and Technology, Nanjing, Jiangsu, China

[4]Tianjin Key Laboratory for Oceanic Meteorology, Tianjin Institute of Meteorological Science, Tianjin, China

[5]Department of Atmospheric and Oceanic Sciences and Institute of Atmospheric Sciences, Fudan University, Shanghai, China

*Correspondence to yang.yang@nuist.edu.cn and hailong.wang@pnnl.gov

[#]These authors contributed equally: Yang Yang, Lili Ren

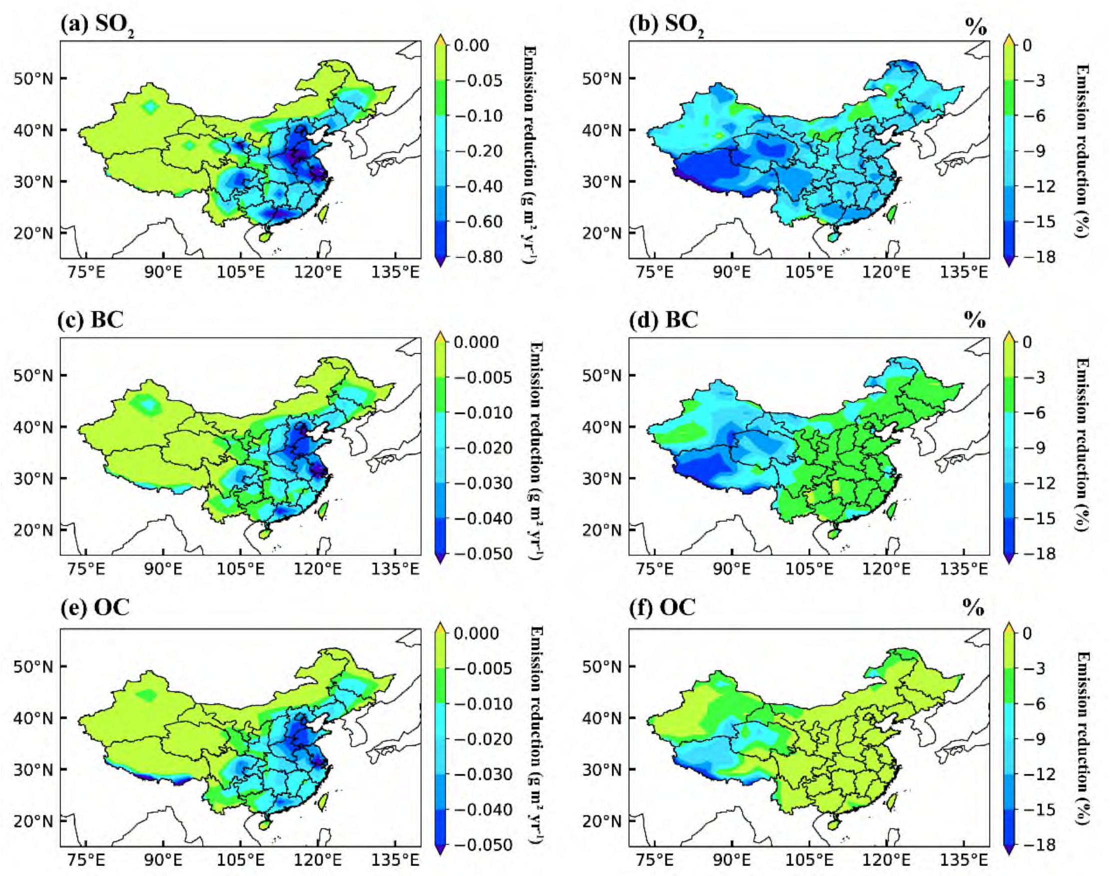

Supplementary Figure 1. Reductions in aerosols and precursor emissions during COVID-19. Spatial distribution of absolute (left, g m^2 yr^{-1}) and percentage (right, %) changes in (a, b) sulfur dioxide (SO_2), (c, d) black carbon (BC) and (e, f) organic carbon (OC) emissions averaged over June-July (JJ) 2020 for Covid experiments compared to Baseline.

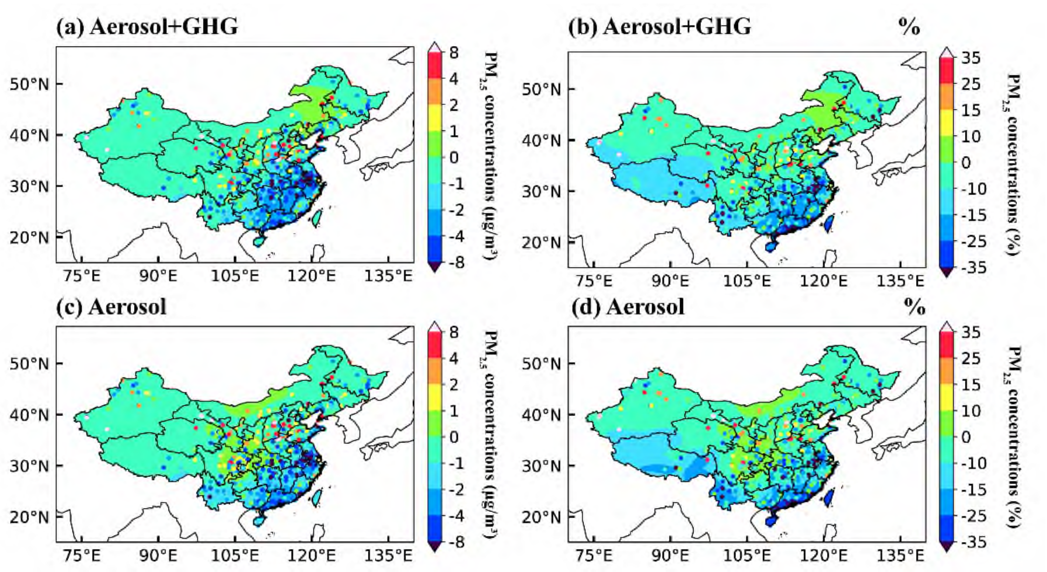

Supplementary Figure 2. Observed and simulated changes in $PM_{2.5}$ concentrations. Spatial distribution of absolute (left, μg m^{-3}) and percentage (right, %) changes in the simulated June-July mean near-surface $PM_{2.5}$ concentrations for Covid_All (a, b) and Covid_Aero (c, d) compared to Baseline (contours), as well as the changes in observed June-July $PM_{2.5}$ concentrations in 2020 compared to 2019 (dots). Only changes in $PM_{2.5}$ observations that are larger than 1.5 σ of the corresponding values over 2015–2019 are shown.

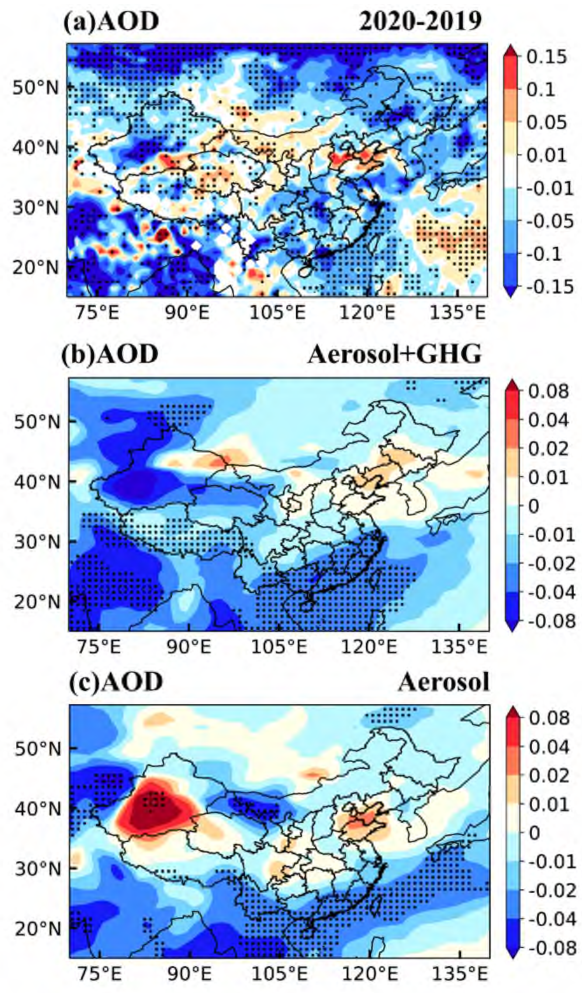

Supplementary Figure 3. Changes in AOD due to COVID-19 emissions reductions. (a) Spatial distribution of changes in 550 nm AOD in summer 2020 compared to 2019 derived from MODIS products using Dark Target and Deep Blue algorithm. Spatial distribution of changes in 550 nm AOD for (b) Covid_All and (c) Covid_Aero compared to Baseline. The stippled areas in (a) indicate that changes are larger than 1.5 σ over 2015–2019 and stippled areas in (b) and (c) indicate statistically significant differences at the 90% confidence level based on a two-tailed Student's t test.

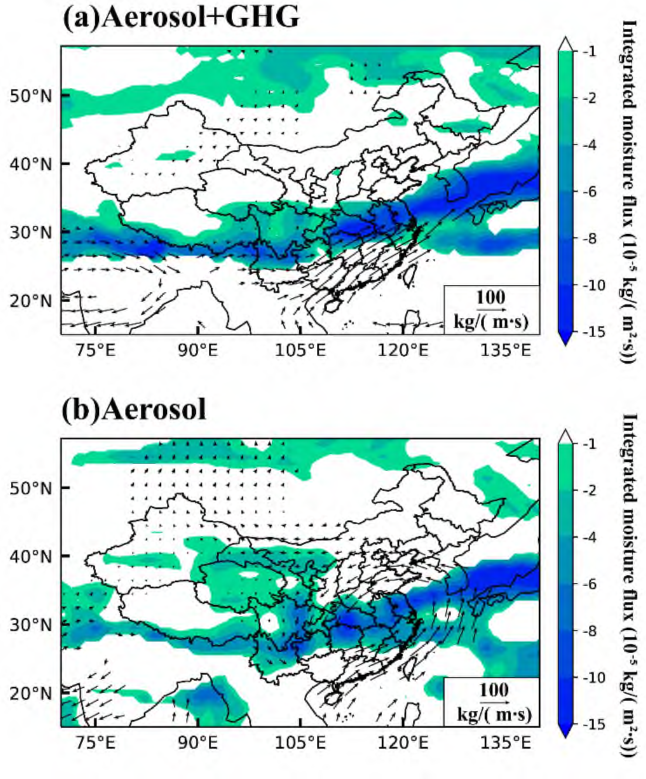

Supplementary Figure 4. Changes in vertically integrated moisture flux and its divergence due to COVID-19 emissions reductions. The anomalies of vertically integrated June-July mean moisture flux (100 kg m^{-1} s^{-1}, vector) and moisture flux divergence (10^{-5} kg m^{-2} s^{-1}, shaded) estimated from Covid_All and Covid_Aero relative to Baseline. Only water vapor flux changes that are statistically significant at the 90% confidence level are shown in (a) and (b).

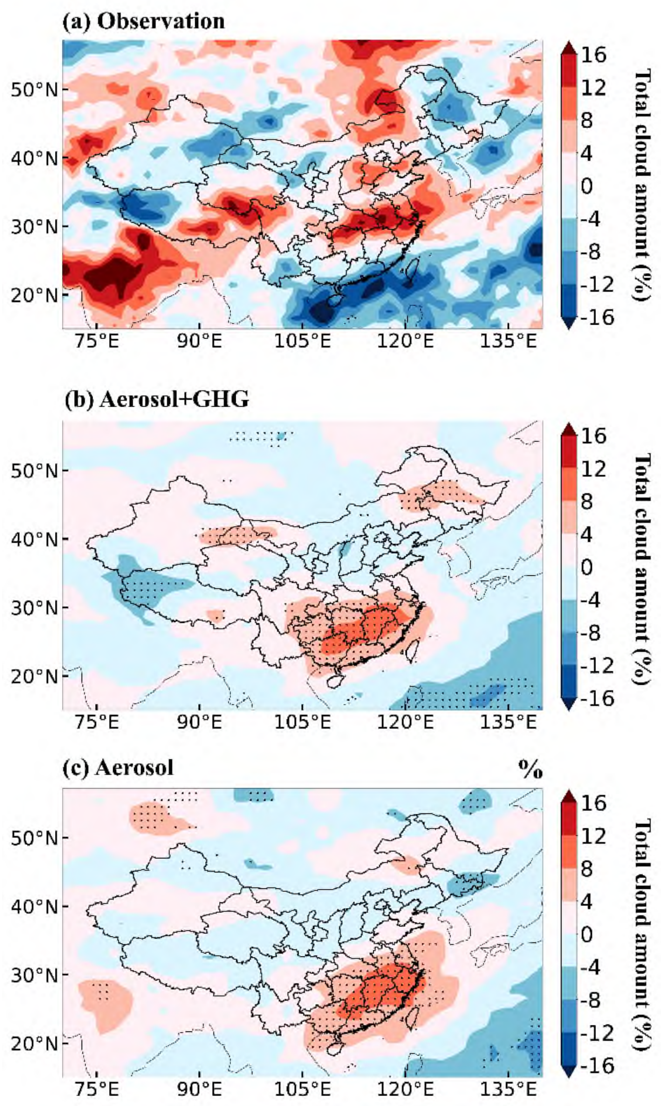

Supplementary Figure 5. Observed and simulated changes in total cloud amount. (a) Observed anomalies of JJ mean total cloud amount (%) in 2020 with respect to the same period of 2019 from MODIS retrievals. (b) and (c) are same as (a) but for simulated total cloud amount changes from in Covid_All and Covid_Aero, respectively, compared to Baseline. The stippled areas in (b) and (c) indicate statistically significant differences at the 90% confidence level based on a two-tailed Student's t test.

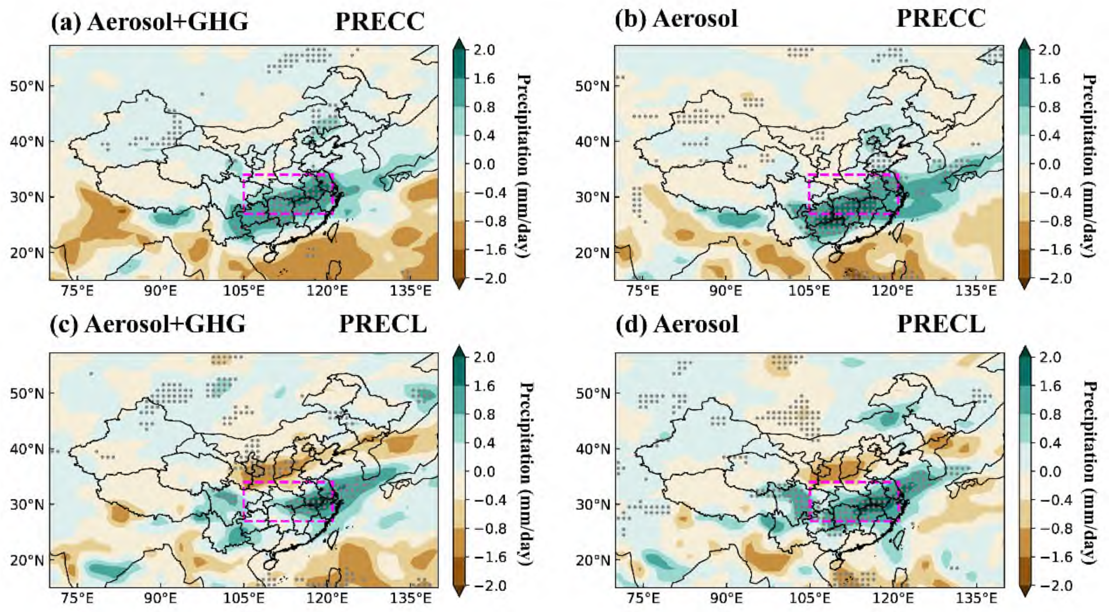

Supplementary Figure 6. Changes in convective and large-scale precipitation. Spatial distribution of changes in JJ (a, b) convective (PRECC) and (c, d) large-scale (PRECL) precipitation rate (mm day^{-1}) for Covid_All (left) and Covid_Aero (right), compared to the Baseline simulation. The stippled areas indicate statistically significant differences at the 90% confidence level based on a two-tailed Student's t test.

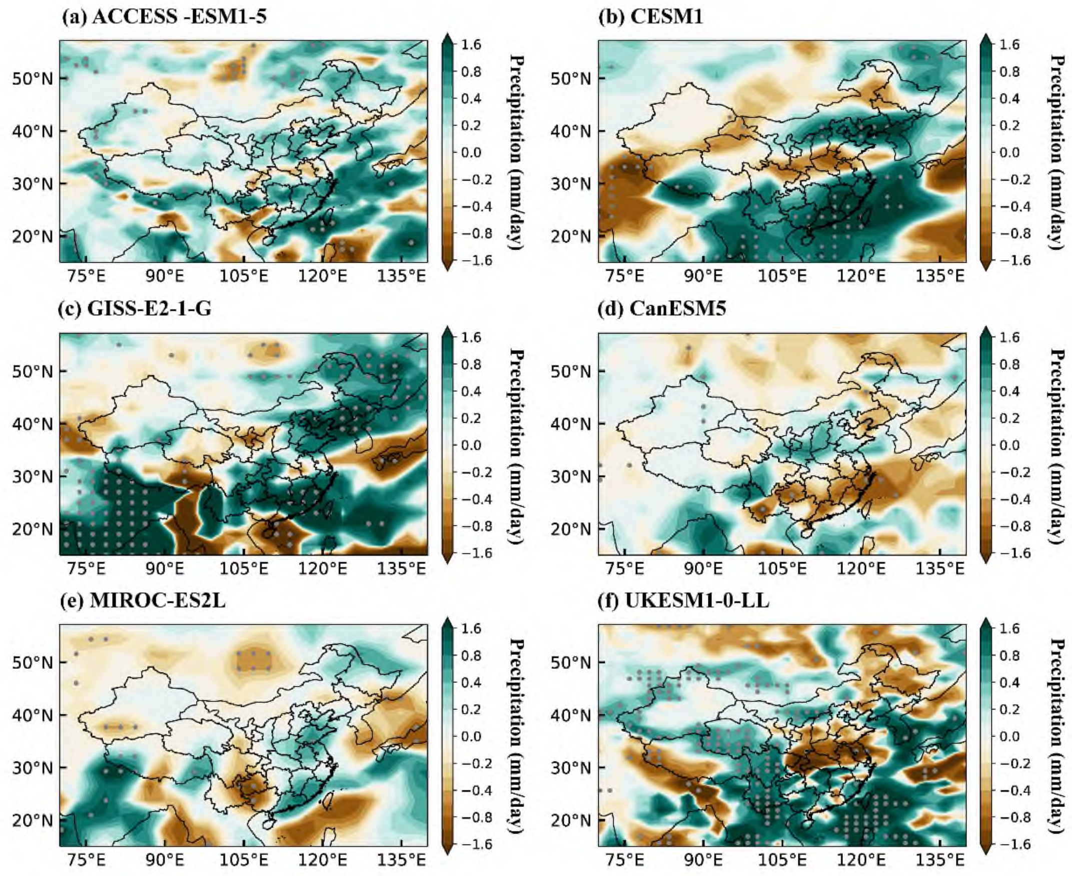

Supplementary Figure 7. Extreme precipitation in China simulated by CovidMIP models. Spatial distribution of changes in June-July precipitation rate (mm day^{-1}) for Covid_All, compared to Baseline, simulated in (a) ACCESS -ESM1-5, (b) CESM1, (c) GISS-E2-1-G, (d) CanESM5, (e) MIROC-ES2L and (f) UKESM1-0-LL. The stippled areas indicate statistically significant differences at the 90% confidence level based on a two-tailed Student's t test. Only models with available ensemble members equal or large than 10 are used.

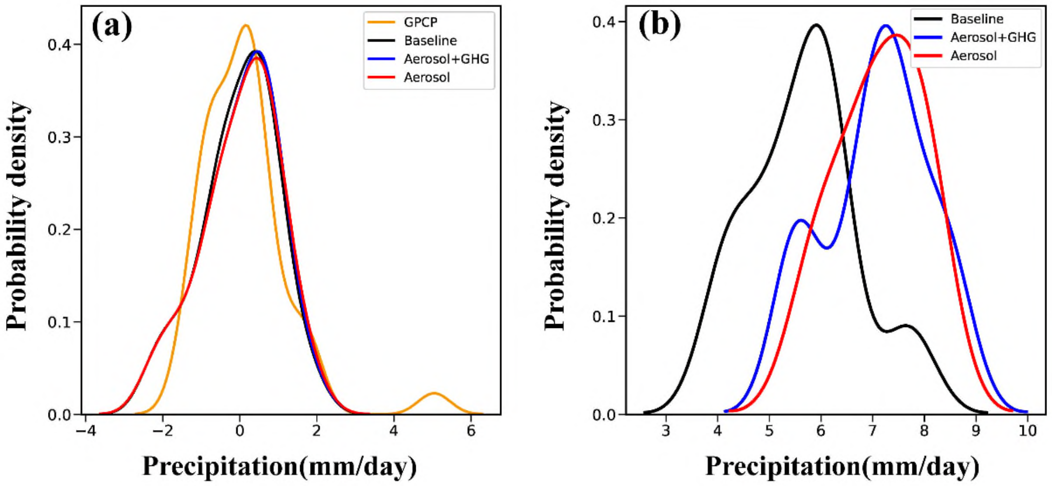

Supplementary Figure 8. Probability density function (PDF) of June-July mean precipitation over eastern China. (a) PDF of June-July mean precipitation anomalies over eastern China (27–34°N, 105–121°E) during 1979–2020 from Global Precipitation Climatology Project (GPCP) (orange line), Baseline (black line), Covid_All (blue line) and Covid_Aero (red line). Note that modeling data over 1979–2019 are from historical (1979-2014) and SSP2-4.5 (2015-2019) experiments in Coupled Model Intercomparison Project Phase 6 (CMIP6) and are the same for the three simulations. (b) PDF of June-July mean precipitation over eastern China from 10 ensemble members of three E3SM experiments.

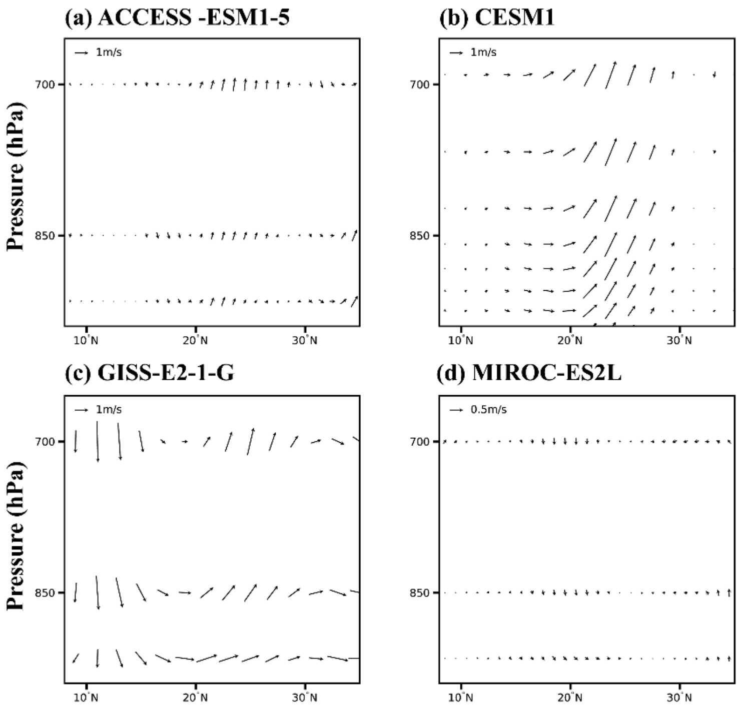

Supplementary Figure 9. Changes in vertical profiles of winds simulated by other CovidMIP models. Changes in June-July mean meridional wind (m s^{-1}, vectors), pressure velocity (Pa s^{-1}, vectors) multiplied by –100 from Covid_All, compared to Baseline, simulated in (a) ACCESS-ESM1-5, (b) CESM1, (c) GISS-E2-1-G and (d) MIROC-ES2L. Only four models having these variables available are used. Note that the 1 m/s reference is used in (a), (b) and (c) and 0.5 m/s reference is used in (d).

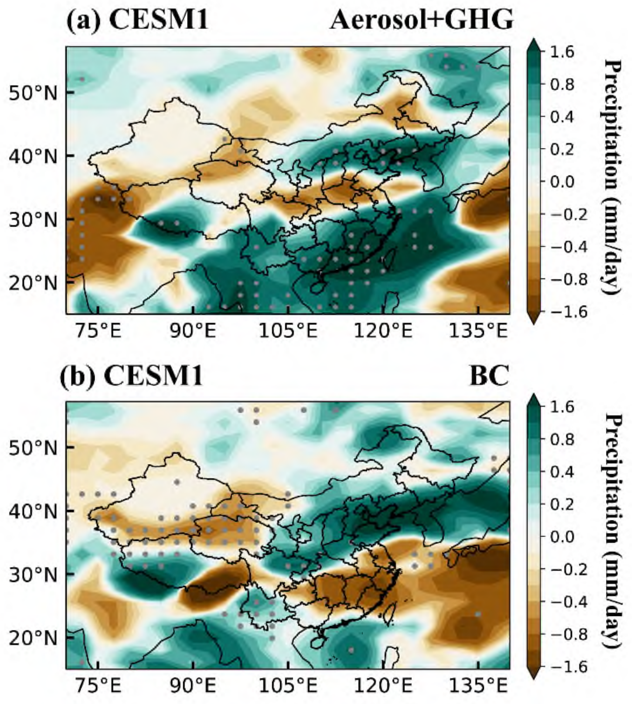

Supplementary Figure 10. Extreme precipitation contributed by all emissions reductions and absorbing aerosol. Spatial distribution of changes in JJ precipitation rate (mm day^{-1}) for (a) Covid_All and (b) a test ensemble simulation with only black carbon emission reduction, compared to Baseline, simulated in CESM1. The stippled areas indicate statistically significant differences at the 90% confidence level based on a two-tailed Student's t test.
